# Supplementary material for: Gain-of-function cardiomyopathic mutations in RBM20 rewire splicing regulation and re-distribute ribonucleoprotein granules within processing bodies
Source: Nat Commun. 2021 Nov 3;12:6324. doi: 10.1038/s41467-021-26623-y (PMC8566601; doi:10.1038/s41467-021-26623-y)
Supplement: Supplementary file 1 — Supplementary Information [file 41467_2021_26623_MOESM1_ESM.pdf]

SUPPLEMENTARY INFORMATION

Gain-of-function cardiomyopathic mutations in RBM20 rewire splicing regulation and re-distribute ribonucleoprotein granules within processing bodies

Fenix and Miyaoka *et al*.

SUPPLEMENTARY METHODS

ddPCR assay to detect the WT, R636S, and R636S+SM alleles

The composition of the premixtures of allele-specific TaqMan probes and primers was 5 μM of an allele-specific FAM or VIC TaqMan MGB probe (Thermo Fisher Scientific), 18 μM of a forward primer and 18 μM of a reverse primer (Integrated DNA Technology) in water. To detect point mutagenesis, we mixed the following reagents in 0.2 ml PCR 8-tube strips: 4 μl water, 12.5 μl 2× ddPCR Supermix for probes (Bio-Rad), 1.25 μl R636S+SM FAM probe and primer premixture, 0.625 μl WT VIC probe and primer premixture, 0.625 μl R636S FAM probe and primer premixture, and 5 μl (50–150 ng) genomic DNA solution (25 μl total volume). The conditions for droplet generation, thermal cycling, and data analysis for RBM20 R636S mutagenesis with the ddPCR system were described before (ref. Miyaoka nat methods). As the R636S+SM FAM probe had a higher concentration than the WT FAM probe, the signal of the R636S+SM allele was distinguishable from that of the WT allele (**Supplementary Figure 1**). Cell populations with a higher frequency of the R636S+SM allele and a lower frequency of the WT allele were enriched by sib-selection until the RBM20 R636S Homo iPS cell clone was isolated.

Oligonucleotide donor DNA used in the present study

RBM20 R636S	ACAGATATGGCCCAGAAAGGCCGCGGTCTAGTAGTCCGGTGAGCCGGTCACTCTCCCCGA
RBM20 R636S+SM	CACAGATATGGCCCAGAAAGGCCGCGGTCAAGTAGTCCGGTGAGCCGGTCACTCTCCCCG

The R636S point mutation and the S635S silent mutation (SM) sites are underlined and double underlined, respectively.

gRNAs used in the present study

1	gCCATATCTGTGAGGGAGCCAAGG
2	gAAGGCCGCGGTCTCGTAGTCCGG

These two gRNAs were used as a dual Cas9 nickase system. RBM20 gRNA-2 specifically targeted the WT allele in R636S Het iPS cells due to the nucleotide difference at the R636S point mutation site, which is underlined. The PAM sequences are double underlined.

Probe-primer sets used in the present study

	Sequence	Fluor-quencher	Final concentration
Forward primer	TGTGAAGATTCTAAATCCTGCTCCTT		900 nM
Reverse primer	AGGAGGTGAAGCTGGGAGTGT		900 nM
WT probe	CCGCGGTCTCGTAG	VIC	125 nM
R636S probe	CGGTCTAGTAGTCC	FAM	125 nM
R636S+SM probe	CCGCGGTCAAGTAG	FAM	250 nM

The R636S point mutation and the S635S silent mutation (SM) sites are underlined and double underlined, respectively.

Supplementary Figure 1

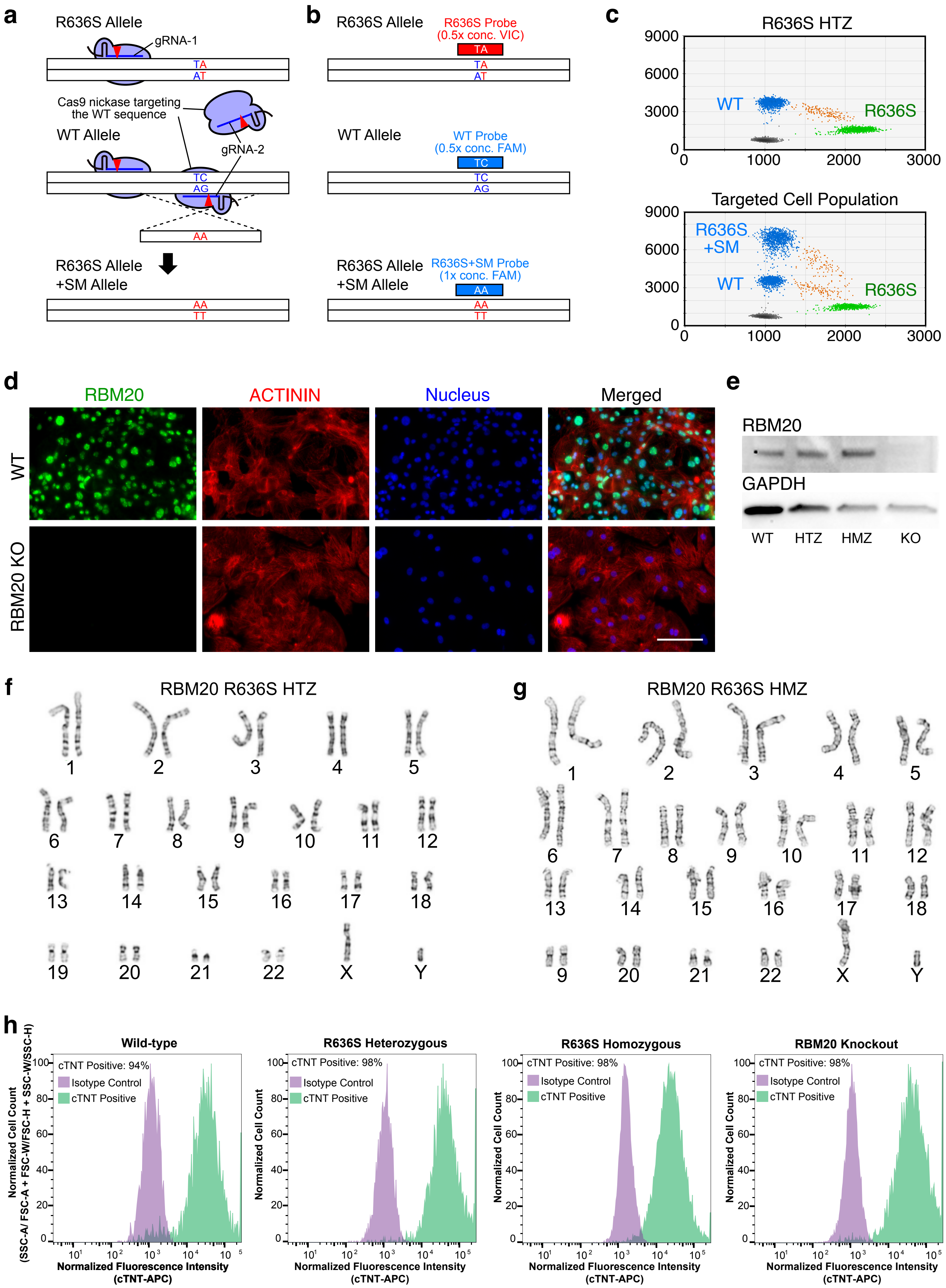

Supplementary Figure 1. **Generation of RBM20 mutant iPSC lines by genome-editing.**

a) Dual Cas9 nickase design to target the WT allele in R636S HTZ iPS cells to generate R636S HMZ cells. RBM20 gRNA-2 is specific to the RBM20 WT allele, so that the R636S allele could not be targeted. Recombination between the WT allele and the donor oligonucleotide with the R636S and the S635S silent mutation resulted in R636S HMZ cells that have the R636S allele and the R636S+SM allele. b) Allele specific probe design and concentration to detect the three alleles by ddPCR. To distinguish the R636S+SM allele from the other two alleles, the R636S+SM probe had two times higher concentration than the other two probes. c) ddPCR analysis to simultaneously detect the three alleles. The parental RBM20 R636S HTZ iPS cells, and a cell population targeted to generate the R636S HMZ iPS cell line were analyzed with the probes shown in B). The R636S+SM allele-positive droplets were observed in the targeted cells as FAM strongly positive population. Cell populations with a higher frequency of the R636S+SM allele and a lower frequency of the WT allele were enriched to isolate the R636S HMZ iPS cell clone. d) Immunofluorescent staining confirming depletion of RBM20 in the 8-bp Del HMZ iPSC-CMs. RBM20 (green) and ACTININ (Red) were visualized in WT and the 8-bp Del HMZ iPSC-CMs. The nuclei (blue) were stained with DAPI, and the merged images are also shown. Scale bar; 100 µm. e) Western blot showing loss of RBM20 protein in RBM20 KO iPSC-CMs. f and g) Karyotypes of R636S HTZ (e) and HMZ (f) iPS cell clones. Both lines maintained a normal male karyotype. h) Flow cytometry demonstrates RBM20 mutant iPSCs successfully differentiate into iPSC-CMs as assessed via cTnT stain following sodium lactate purification. Gating strategy for cTnT positive cells (i.e., cardiomyocytes): 1) side scatter area (SSC-A) + forward scatter area (FSC-A), 2) forward scatter width (FSC-W) + forward scatter height (FSC-H), 3) side scatter width (SSC-W) + side scatter height (SSC-H), 4) APC intensity. Source data are provided as a Source Data file.

Supplementary Figure 2

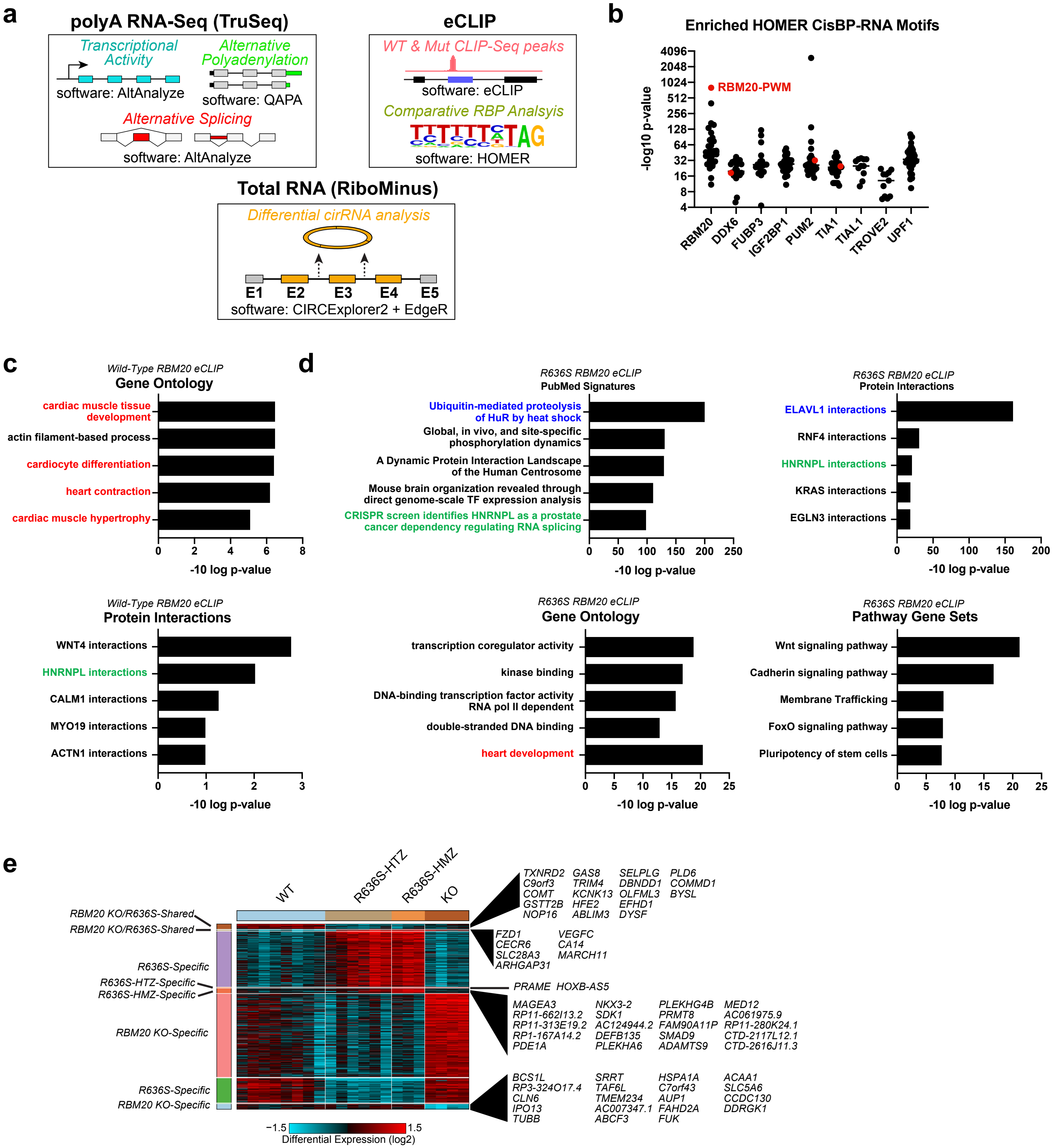

Supplementary Figure 2. **RBM20 mutant bound transcripts and functional impact.**

a) Graphical overview of the "omics" integrative analysis strategy and analytical approaches to identify molecular impacts of RBM20 mutation or deletion. b) Enrichment of RBM20 binding sites among RBPs with RBM20-mutant shared eCLIP peaks by HOMER using RNA recognition Elements from the CisBP-RNA database. c-d) Gene-set enrichment of analyses with the software ToppFun of genes with reproducible eCLIP peaks in wild-type (c) and R636S-HMZ (d) iPSC-CMs. e) MarkerFinder gene expression heatmap from Fig. 5, with specific genes noted for minor expression patterns.

Supplementary Figure 3

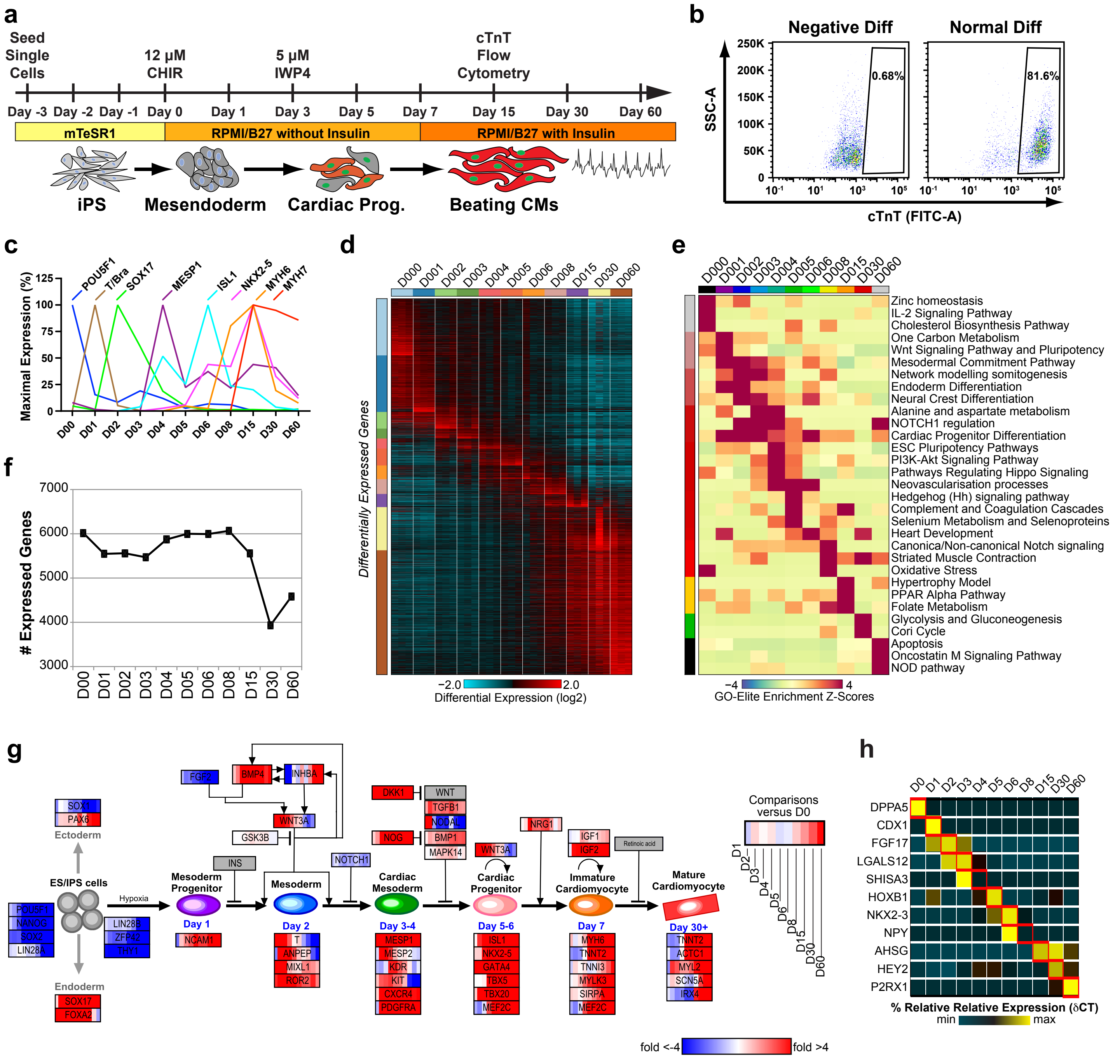

Supplementary Figure 3. **Stage-specific transcriptomic differences in cardiomyocyte differentiation**. a) The GiWi protocol was used to obtain day 30 differentiated cardiomyocytes which were assessed at day 15 for negative and normal differentiated samples stained with cTnT by flow-cytometry. b) Differentiation efficiencies were measured by cTnT staining at day 15 by flow cytometry. The Normal Diff sample displays a clear enhancement in fluorescence intensity compared to the Negative (no CHIR or IWP4 treatment) Diff marking cTnT+ cells. c) TaqMan analysis shows the sequential progression from iPS cells (POU5F1) into functional CMs (NKX2-5, MYH6 & MYH7). Relative gene expression is graphed as a percentage of the maximal expression observed. d) The total number of detected genes (protein-coding and ncRNA) expressed with an RPKM > 3 and at least 50 reads/gene at each time-point of differentiation. e) Heatmap of all differentially expressed genes (fold>1.5 and eBayes two-sided t-test $p<0.05$, FDR corrected) for each timepoint for versus day 0, ordered by the software MarkerFinder. f) Heatmap of the predominant WikiPathways associated with top-200 time-point specific marker genes, based on Z-score enrichment (GO-Elite software). g) Temporal changes in gene expression (log2 fold change) across time-points of cardiac differentiation, visualized in the context of prior defined cardiac differentiation markers in PathVisio (WikiPathways:WP2406). h) Heatmap of qPCR validation results (delta CT values) of the top-predicted RNA-Seq marker genes for each time-point.

Supplementary Figure 4

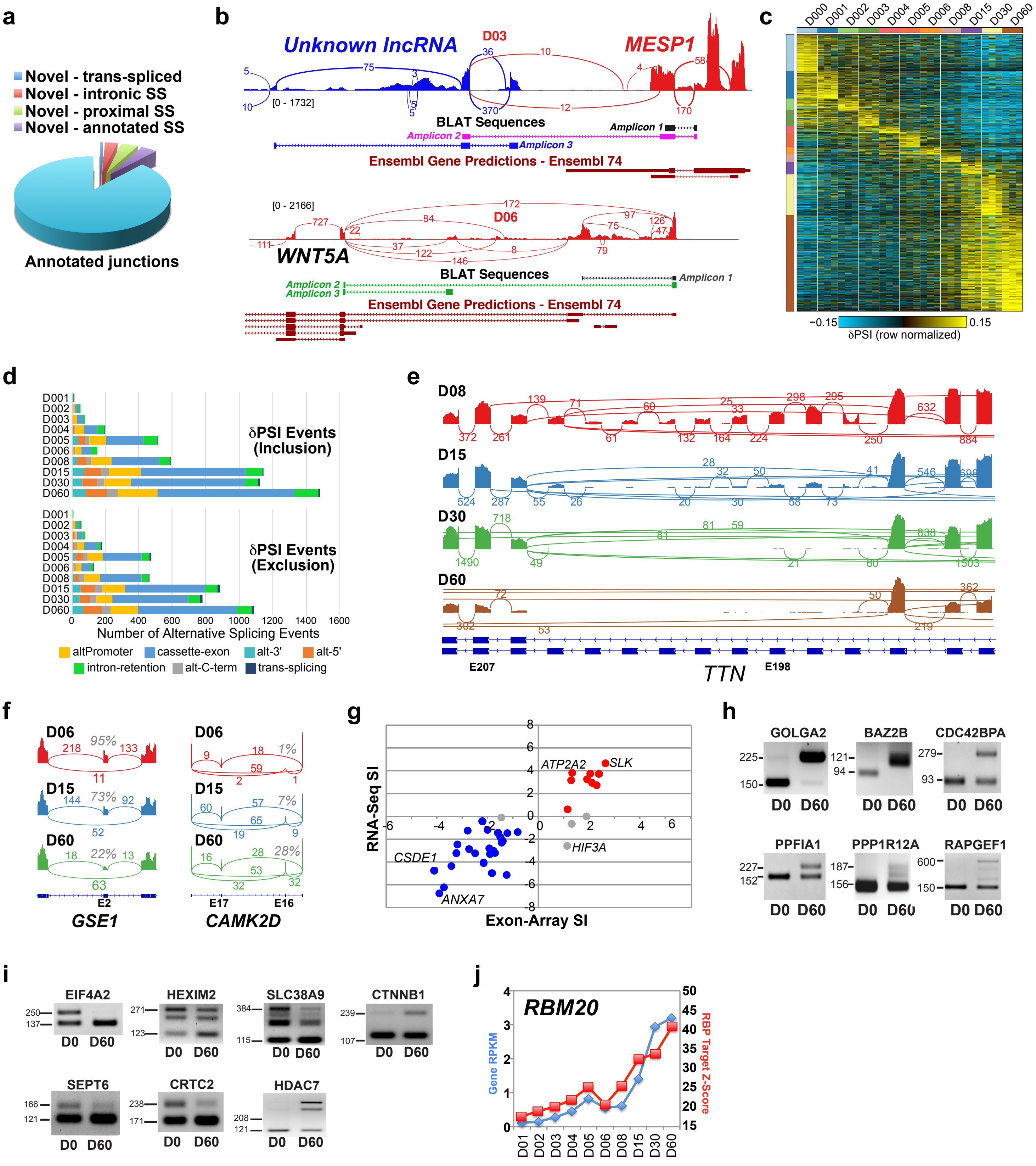

Supplementary Figure 4. **Validation of RBM20-mediated splicing during iPSC-CMs differentiation.** a) Extent of expressed known and novel exon-exon junctions (at least 5 reads in 3 or more samples) observed from all cardiac differentiation RNA-Seq samples. Trans-spliced events are between two adjacent or distant genes, proximal splice-sites (SS) are those occurring within 50nt of a known splice site and intronic are those occurring >50nt away. b) Genes with novel exons were identified from AltAnalyze and verified by targeted amplification (amplicon) and Sanger sequencing. BLAT aligned sequences are derived from the consensus of forward and reverse primer amplifications. MESP1 splicing to an unknown (UNK) lncRNA on the reverse strand. One confirmed mRNA downstream for this lncRNA is shown in blue (amplicon 3), with a trans-splicing isoform produced between MESP1- and this lncRNA in purple (amplicon 2). Two novel N-terminal alternative isoforms of the gene WNT5A, with an alternatively excluded exon 2 (amplicon 2) and a novel alternative promoter regulated exon (amplicon 3) in green on the reverse strand. c) Heatmap of all differential splicing events (δPSI>0.1 and eBayes two-sided t-test p<0.05, FDR corrected) for each timepoint for versus day 0, ordered by the software MarkerFinder. Associated statistics for each splicing event are provided in **Supplementary Data 8**. d) Incidence of alternative splicing for exon-inclusion (top) and exon-exclusion for each iPSC-CM differentiation versus day 0, by Percent Spliced-In analysis. e) Heatmap of the top-200 unique splicing-events for all differentiation time-points. E) SashimiPlot of TTN exons spliced-out during distinct phases of iPSC-CM commitment. f) SashimiPlots illustrating the temporal regulation of novel (GSE1) and known (CAMK2D) RBM20 target exons by alternative splicing. Curved lines indicate splice-junctions with associated aligned reads. The percentage indicates the amount of relative exon-inclusion. g) Comparison of previously identified day 40 cardiac differentiation associated alternative splicing events (Salomonis et al. PLoS Comput Biol. 2009) to similar computed exon-level splicing-index values from iPSC-CM day 30 RNA-Seq. Shown are the 41 out of 47 expressed exons that could be comparably mapped to AltAnalyze annotated exons are shown. red=positive SI fold (exon-inclusion in hESC), blue=negative SI fold (exon-inclusion in CM), grey=disagreeing. h-i) Representative RT-PCR images for novel detected iPSC-CM differentiation splicing events, denoting the expected size of each amplicon. Example novel RBM20

target splicing-events, evidenced from gene-edited iPSC-CMs, with evidence of differentiation regulation are separately called out in panel i. j) Gene expression level of RBM20 throughout differentiation (blue points), co-visualized with GO-Elite Z-score enrichment results for alternatively spliced exons in differentiation (versus day 0) relative to RBM20-mutant patient (dilated cardiomyopathy) versus control splicing events. Source data are provided as a Source Data file.

Supplementary Figure 5

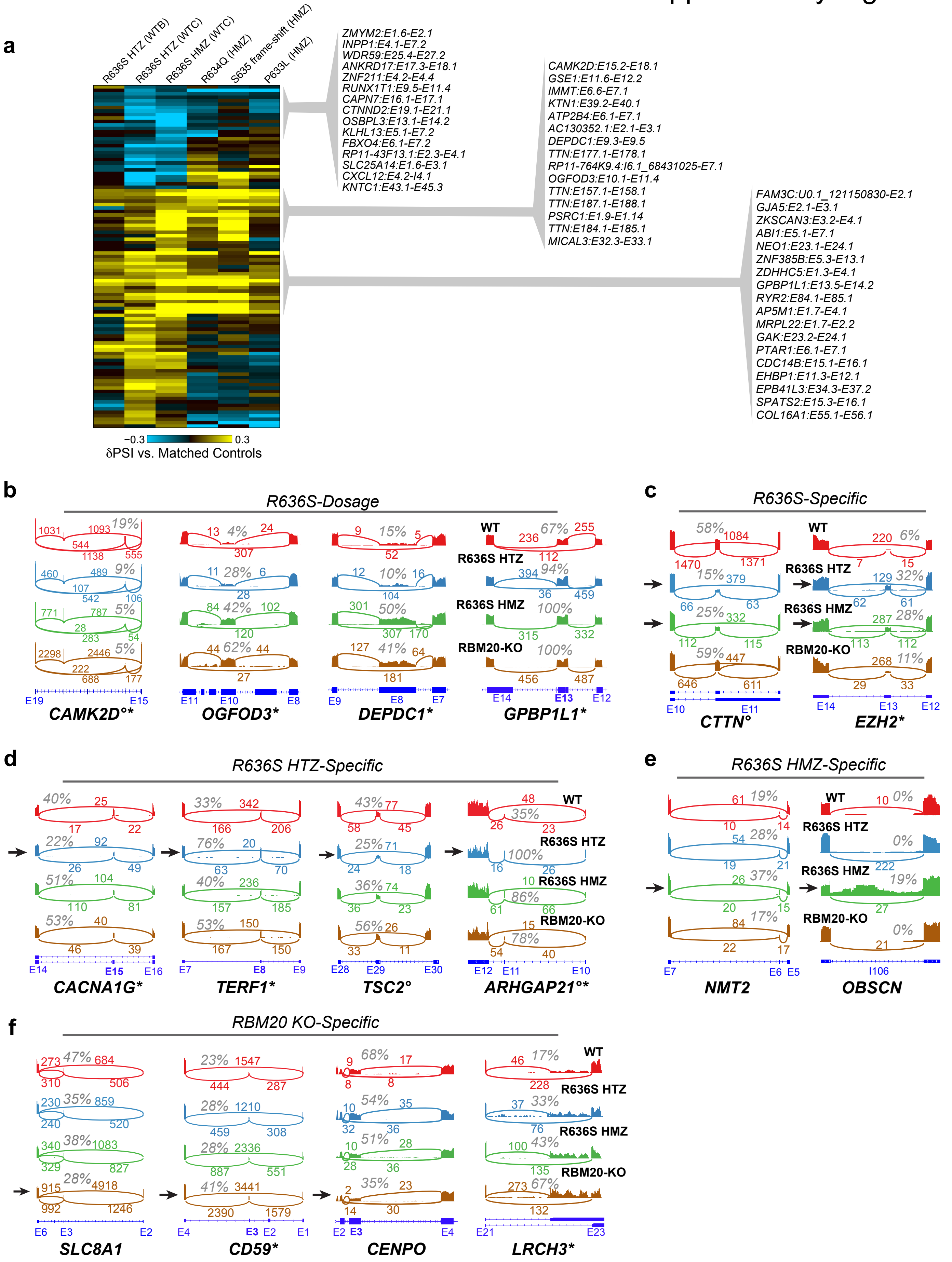

Supplementary Figure 5. Visualization and verification of specific iPSC-CM RBM20 splicing events.

a) Heatmap of shared splicing patterns observed in independently RBM20 edited iPSC-CM lines (δPSI vs. matched control lines). The R636S HTZ mutation was edited into the WTB line, whereas independently edited HMZ iPSC-CMs (including HMZ S635 frame-shift), were reanalyzed from Briganti et al. b-f) SashimiPlots of example splicing events from Fig. 4c, associated with the indicated patterns of unique or shared regulation. * = verified splicing event patterns inferred from the independently edited iPSC-CMs. ° = verified splicing event from R636S HTZ edited pig hearts.

Supplementary Figure 6

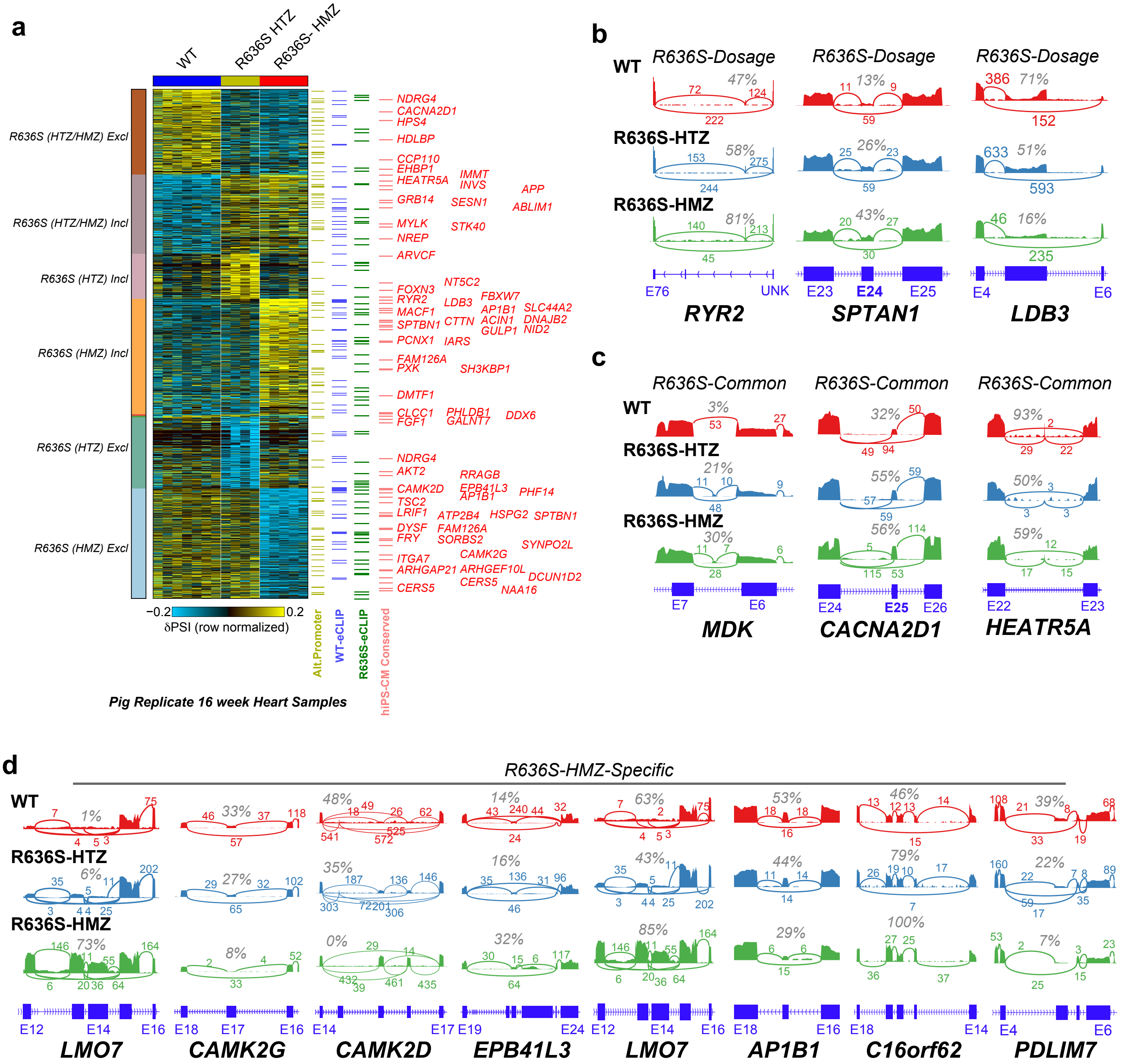

Supplementary Figure 6. **Porcine 16-week neonatal heart R636S-mutant regulated splicing events.**

a) Heatmap of the predominant patterns of alternative splicing in the pig heart for significantly differential events (δPSI>0.1 and eBayes two-sided t-test p<=0.05). See Fig. 4c for a detailed description of the overlapping eCLIP and human-conserved splicing event predictions. Genes for conserved hiPS-CM RBM20 mutant splicing events are displayed to the right. Associated statistics for each splicing event are provided in **Supplementary Data 20**. b-d) Example SashimiPlots of R636S-regulated splicing events in pigs that correspond to conserved and non-conserved events. Specifically, R636S events with observed dosage dependent splicing (b), R636S dosage-independent (c) and R636S-HMZ-specific splicing events.

Supplementary Figure 7

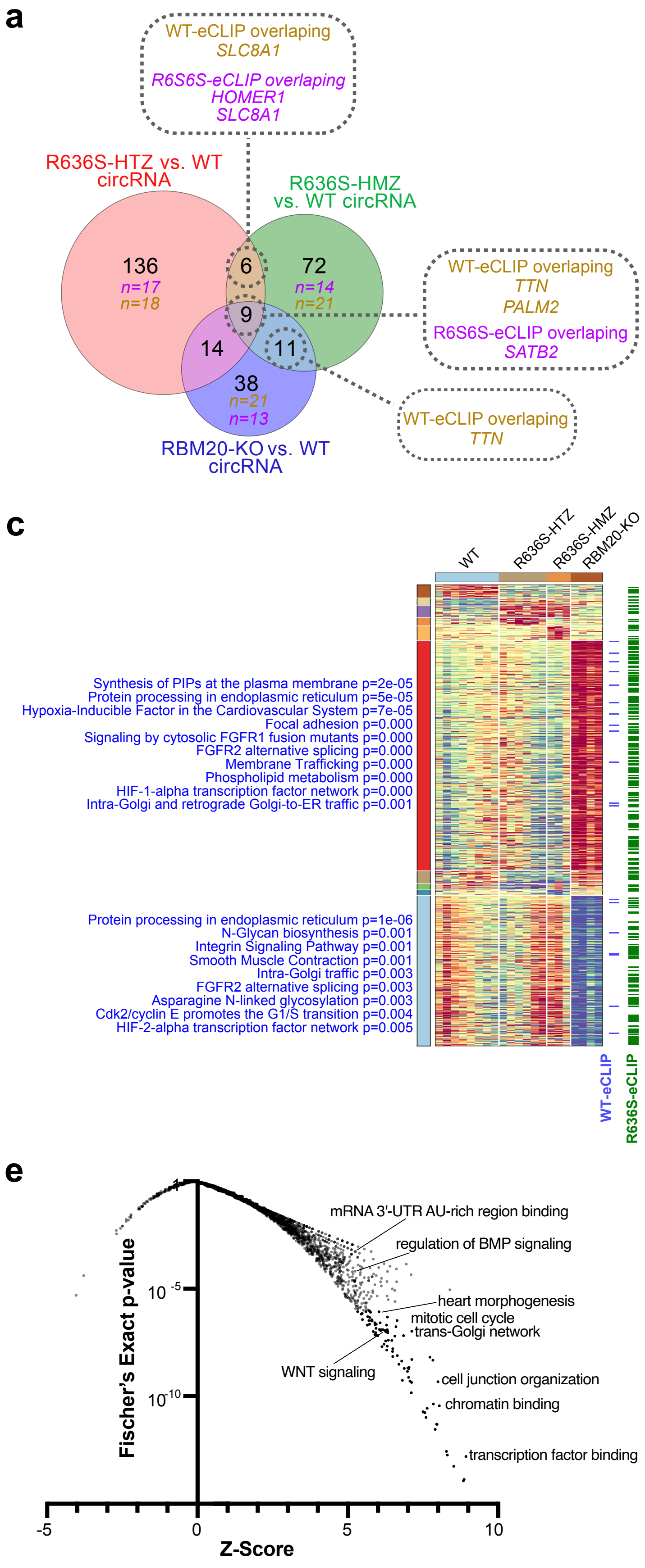

Supplementary Figure 7. **Impact of RBM20 mutation on circular RNA and alternative polyadenylation**.

a) Venn diagram of differentially regulated circRNAs, comparing R636S mutants or RBM20 KO to wild-type RiboMinus RNA-Seq sample groups (EdgeR $p<0.1$). circRNA genes with evidenced eCLIP peak genomic overlaps are denoted, based on the eCLIP source (mutant or wild-type). The number of circRNAs with eCLIP overlaps are denoted in each Venn circle (purple = R636S eCLIP, gold = WT eCLIP). b) Venn diagram of differentially expressed circRNAs that also have genomic overlapping alternative splicing events for the indicated sample-group comparisons. Such events represent coordinately regulated circRNAs and alternative-splicing events. The majority of such events are associated with a single gene (*TTN*), that are associated with multiple independent splicing events. c) Heatmap of alternative polyadenylation (APA) 3′ UTRs, organized using the software MarkerFinder into the predominant patterns of regulation. Gene-set enrichment results (GO-Elite) for each cluster are shown to the left of the heatmap (ToppFun pathway collection). Note that for each APA event, at least one reciprocal redundant APA event may also be reported (e.g., short form, long form). Any reproducible eCLIP peaks within the gene body of APA impacted genes are shown are indicated to the right of the heatmap. d) Venn diagram of genes with APA events significantly up-regulated in RBM20 KO versus R636S HMZ iPSC-CMs (relative APA ratio differences > 0.1 and eBayes two-sided t-test $p<0.05$, unadjusted) and genes with observed R636S eCLIP peaks. e) Gene Ontology enrichment analysis (GO-Elite) for the 534 overlapping RBM20 KO and R636S eCLIP peaks from panel d (Fisher Exact test $p<0.05$, raw). Source data are provided as a Source Data file.
