# Supplementary material for: Gain-of-function cardiomyopathic mutations in RBM20 rewire splicing regulation and re-distribute ribonucleoprotein granules within processing bodies
Source: Nat Commun. 2021 Nov 3;12:6324. doi: 10.1038/s41467-021-26623-y (PMC8566601; doi:10.1038/s41467-021-26623-y)
Supplement: Supplementary file 3 — Description of Additional Supplementary Files [file 41467_2021_26623_MOESM3_ESM.pdf]

Description of additional supplementary files

Title: Supplementary Movie 1

Description: WT iPSC-CMs beat spontaneously on Multi-electrode Array (MEA) plates. Black structures are electrodes. Video played at real time, 19.9 fps. Video length = 10 seconds, magnification = 20x. Brightfield microscopy.

Title: Supplementary Movie 2

Description: 3D-EHTs cast from iPSC-CMs spontaneously beat in culture. Flexible post (left) is moved during contraction while stiff glass post (right) remains motionless. Video played at real time, 66 fps. Video length = 5 seconds, magnification = 2x. Brightfield microscopy.

Title: Supplementary Movie 3

Description: 3D-EHTs cast from iPSC-CMs can be electrically paced. Tissue has been paced at 2 Hz. Video is played at real time, 66.6 fps. Video length = 3 seconds, magnification = 2x. Brightfield microscopy.

Title: Supplementary Data File 1.

Description: RNA-Sequencing Dataset Metrics.

Title: Supplementary Data File 2.

Description: RBM20 wild-type iPSC-CM eCLIP IDR peaks (produced from the standard ENCODE pipeline).

Title: Supplementary Data File 3.

Description: RBM20 R636S-HMZ iPSC-CM eCLIP IDR peaks (produced from the standard ENCODE pipeline).

Title: Supplementary Data File 4.

Description: RBM20 R636S-HMZ iPSC-CM eCLIP IDR peaks overlapping with FUS-S5252 peaks (produced from the standard ENCODE pipeline).

Title: Supplementary Data File 5.

Description: ToppFun and GO-Elite gene-set enrichment (Fisher Exact test p-value < 0.05 and number of genes changed > 3) for genes associated with RBM20-mutant/FUS-mutant overlapping peaks.

Title: Supplementary Data File 6.

Description: Differentially expressed genes (eBayes two-sided t-test p<0.05, FDR corrected) for RBM20 KO vs. RBM20 R636S (HTZ+HMZ).

Title: Supplementary Data File 7.

Description: Differentially expressed genes from all genotype pairwise comparisons (fold>1.5, eBayes two-sided t-test p<0.05, FDR corrected) organized into predominant MarkerFinder patterns.

Title: Supplementary Data File 8.

Description: Differentially expressed genes in differentiating WTB iPSC (fold>2, eBayes two-sided t-test p<0.05, FDR corrected).

Title: Supplementary Data File 9.

Description: GO-Elite gene set enrichment (Fisher Exact test p-value < 0.05 and number of genes changed > 2) for MarkerFinder assigned differentially expressed genes for each pattern (see Extended Data Fig. 3F).

Title: Supplementary Data File 10.

Description: Differential splicing events (eBayes two-sided t-test p<0.05, FDR) from MultiPath-PSI for WTB iPSC-CM time-points versus Day 0 (iPSC).

Title: Supplementary Data File 11.

Description: Verification of RT-PCR prior validated day 40 hESC-CM vs. day 0 hESC alternative splicing events predicted from exon-array analysis (Salomonis et al. PLoS CB 2009) in day 30 iPSC-CM vs. day 0 iPSC from WTB RNASeq (splicing-index value comparison) (results not statistically filtered).

Title: Supplementary Data File 12.

Description: Validation of novel iPS-CM differentiation regulated splicing events by RT-PCR (comparison of amplicon molecular weights and abundance).

Title: Supplementary Data File 13.

Description: High confidence alternative splicing events for a DCM patient with RBM20-S635A AltAnalyze evidenced exon-specific events from both the exon-exon junction ASPIRE comparison algorithm and the exon-level splicing-index algorithm. These algorithms were applied as MultiPath-PSI requires replicate samples for both compared groups.

Title: Supplementary Data File 14.

Description: Alternative splicing differences predicted from RNA-Seq comparison of WTC iPS-CMs with R636S-HTZ vs. RBM20-WT (limma two-sided p.FDR < 0.1, dPS>0.1).

Title: Supplementary Data File 15.

Description: Alternative splicing differences predicted from RNA-Seq comparison of WTB iPS-CMs with R636S-HTZ vs. RBM20-WT (n=2) (no statistical filtering).

Title: Supplementary Data File 16.

Description: Alternative splicing differences predicted from RNA-Seq comparison of iPS-CMs with R634Q vs. RBM20-WT (Briganti et al. Cell Reports 2020) (no statistical filtering).

Title: Supplementary Data File 17.

Description: Alternative splicing differences predicted from RNA-Seq comparison of WTC iPS-CMs with R636S-HMZ vs. RBM20-WT (limma two-sided p.FDR < 0.1, dPS>0.1).

Title: Supplementary Data File 18.

Description: Alternative splicing differences predicted from RNA-Seq comparison of WTC iPS-CMs with RBM20 KO vs. RBM20-WT (limma two-sided p.FDR < 0.1, dPS>0.1).

Title: Supplementary Data File 19.

Description: Global splicing-patterns for unique WTC iPSC-CM RBM20 impacted events from MarkerFinder (filtered based by MarkerFinder for events with a Pearson correlation coefficient > 0.5 to each idealized pattern reference).

Title: Supplementary Data File 20.

Description: Alternative splicing differences predicted from RNA-Seq comparison of neontal pig hearts with R636S-HTZ vs. RBM20-WT (limma two-sided p <= 0.01 (raw), dPS>0.1).

Title: Supplementary Data File 21.

Description: GO-Elite enrichment (Fisher Exact test p-value < 0.05 and number of genes changed > 2) of ToppFun Gene-Sets for RBM20 splicing patterns derived from the MarkerFinder analysis.

Title: Supplementary Data File 22.

Description: Differential circRNAs from edgeR (two-sided p<0.1, raw) comparing RBM20 mutants vs. WT.

Title: Supplementary Data File 23.

Description: Differentially expressed alterantive polyadenylation isoforms in differentiating WTC iPSC-CMs (dAPA>0.1, eBayes two-sided t-test p<0.05, FDR corrected).

Title: Supplementary Data File 24.

Description: GO-Elite enrichment of (Fisher Exact test p-value < 0.05 and number of genes changed > 2) ToppFun Gene-Sets for RBM20 APA patterns derived from the MarkerFinder analysis.
