# Supplementary material for: Gain-of-function cardiomyopathic mutations in RBM20 rewire splicing regulation and re-distribute ribonucleoprotein granules within processing bodies
Source: Nat Commun. 2021 Nov 3;12:6324. doi: 10.1038/s41467-021-26623-y (PMC8566601; doi:10.1038/s41467-021-26623-y)
Supplement: Supplementary file 31 — Reporting Summary [file 41467_2021_26623_MOESM31_ESM.pdf]

nature portfolio

Corresponding author(s): Bruce R. Conklin, Charles E. Murry and Nathan Salomonis

Last updated by author(s): 10/18/2021

Reporting Summary

Nature Portfolio wishes to improve the reproducibility of the work that we publish. This form provides structure for consistency and transparency in reporting. For further information on Nature Portfolio policies, see our Editorial Policies and the Editorial Policy Checklist.

Statistics

For all statistical analyses, confirm that the following items are present in the figure legend, table legend, main text, or Methods section.

n/a	Confirmed	
☐	☒	The exact sample size (*n*) for each experimental group/condition, given as a discrete number and unit of measurement
☐	☒	A statement on whether measurements were taken from distinct samples or whether the same sample was measured repeatedly
☐	☒	The statistical test(s) used AND whether they are one- or two-sided<br>*Only common tests should be described solely by name; describe more complex techniques in the Methods section.*
☐	☒	A description of all covariates tested
☐	☒	A description of any assumptions or corrections, such as tests of normality and adjustment for multiple comparisons
☐	☒	A full description of the statistical parameters including central tendency (e.g. means) or other basic estimates (e.g. regression coefficient) AND variation (e.g. standard deviation) or associated estimates of uncertainty (e.g. confidence intervals)
☐	☒	For null hypothesis testing, the test statistic (e.g. *F*, *t*, *r*) with confidence intervals, effect sizes, degrees of freedom and *P* value noted<br>*Give P values as exact values whenever suitable.*
☐	☒	For Bayesian analysis, information on the choice of priors and Markov chain Monte Carlo settings
☐	☒	For hierarchical and complex designs, identification of the appropriate level for tests and full reporting of outcomes
☐	☒	Estimates of effect sizes (e.g. Cohen's *d*, Pearson's *r*), indicating how they were calculated

Our web collection on statistics for biologists contains articles on many of the points above.

Software and code

Policy information about availability of computer code

Data collection	[Flow Cytometry] MACSQuant VYB flow cytometer (Miltenyi Biotec)<br>[Multi Electrode Array] Maestro MEA system (Axion Biosystems)<br>[Structured Illumination Microscopy Image Reconstruction] softWoRX (GE)<br>[3-dimensional Z-stack spinning disk confocal image binarization] Cell Segmenter (Allen Institute) [3-dimensional Z-stack SIM images binarization] Imaris (version 9.7)<br>[Western blot chemiluminescence] ChemiDoc Imaging System (Bio-Rad)

Data analysis

[Flow Cytometry] FlowJo (version 10.8)
[Contractile analysis] Matlab (2020b)
[3-dimensional Z-stack spinning disk confocal image co-localization] Cell Profiler (version 4.0.4)
[RNA binding site analysis] HOMER (version 4.09)
[eCLIP data analysis] https://www.encodeproject.org/eclip/
[eCLIP peak annotation] https://github.com/Yeolab/clipper/
[eCLIP peak comparison] bedtools (version 2.28.0)
[RNA-Seq read alignment] STAR (version 2.4.0)
[RNA-Seq transcriptome analysis] AltAnalyze (version 2.1.4)
[Alternative splicing analyses] Multi Path-PSI (version 2.0)
[Gene-set enrichment] GO-Elite (version 1.2.6) and ToppFun
[Alterantive Polyadenylation analysis] QAPA (version 1.3.2)
[Mixed effects-linear model] LIMMA (version 3.13)
[circRNA read alignment] TopHat2 (version 2.1.1)
[circRNA quantification] CIRCexplorer2 (version 2.3.0)
[Differential circRNA analysis] csbbcompbio (version 3.0)

For manuscripts utilizing custom algorithms or software that are central to the research but not yet described in published literature, software must be made available to editors and reviewers. We strongly encourage code deposition in a community repository (e.g. GitHub). See the Nature Portfolio guidelines for submitting code & software for further information.

Data

Policy information about availability of data

All manuscripts must include a data availability statement. This statement should provide the following information, where applicable:

- Accession codes, unique identifiers, or web links for publicly available datasets
- A description of any restrictions on data availability
- For clinical datasets or third party data, please ensure that the statement adheres to our policy

-RBM20-mutant and wild-type RNA-Seq and eCLIP data (GSE175886). iPSC-CM time-course RNA-Seq data Synapse (https://www.synapse.org/#!Synapse:syn2582579).
-Figures 3-5 and Extended Data Figures 2-7 have associated raw sequencing data corresponding to the above accession numbers.
-All sequencing dataset are provided as open-access in accordance with the data release policies of GEO/SRA and Synapse.

Field-specific reporting

Please select the one below that is the best fit for your research. If you are not sure, read the appropriate sections before making your selection.

☒ Life sciences ☐ Behavioural & social sciences ☐ Ecological, evolutionary & environmental sciences

For a reference copy of the document with all sections, see nature.com/documents/nr-reporting-summary-flat.pdf

Life sciences study design

All studies must disclose on these points even when the disclosure is negative.

Sample size

A minimum of three replicates for RNA-Seq sample groups was used or greater when initial variability was observed by supervised or unsupervised clustering analyses (HOPACH, PCA). Replicate pairs were used for eCLIP, as the standard for all ENCODE eCLIP assays, to identify reproducible peaks. No statistical methods were used to calculate sample size. Sample sizes are sufficient as statistically confident biological phenomena (e.g., expression levels) are reproducible, and verified via complementary measurements (e.g., RT-PCR).

Data exclusions

None noted

Replication

-For alternative splicing RT-PCR validations, at least two biological replicates were analyzed (independently) and were qualitatively consistent.
-Contractility, physiology and RBM20 localization assays were independently replicated in independent assays as indicated in the Methods and Figure legends. RT-PCR to confirm alternative splicing events was performed one time.

Randomization

Isogenic mutations were introduced into a single iPSC-CM line to exclude genetic effects, with verification in independent iPSC-CM lines with the same or other patient mutations. Quantitative analysis was only performed on isogenic lines. That is, mutant lines were only compared to parent line in which mutation was made (i.e., isogenic lines).

Blinding

Only supervised analyses between sample groups with introduced genetic lesions or already defined antibodies were performed in this study. Investigators
were not blinded. Blinding was not necessary, as quantitative measurements were performed in batch on automated analysis pipelines. The sole
exception being figure 2C, where the analysis was performed by hand by AMF. These results are supported via visual analysis (Figure 2B), the analysis pipeline is throughly described in Methods, and all raw data is available upon request.

Reporting for specific materials, systems and methods

We require information from authors about some types of materials, experimental systems and methods used in many studies. Here, indicate whether each material, system or method listed is relevant to your study. If you are not sure if a list item applies to your research, read the appropriate section before selecting a response.

Materials & experimental systems

n/a	Involved in the study
☐	☒ Antibodies
☐	☒ Eukaryotic cell lines
☒	☐ Palaeontology and archaeology
☐	☒ Animals and other organisms
☒	☐ Human research participants
☒	☐ Clinical data
☒	☐ Dual use research of concern

Methods

n/a	Involved in the study
☒	☐ ChIP-seq
☐	☒ Flow cytometry
☒	☐ MRI-based neuroimaging

Antibodies

Antibodies used	RBM20 polyclonal antibody (ThermoFisher, Cat#PA5-58068, 1:500)<br>Mouse anti-human cardiac Troponin T (cTnT) primary antibody (Thermo, MS-295-P, 1:50)<br>Alexa Fluor 488 goat anti-mouse lgG secondary antibody (lnvitrogen, A-11029, 1:100)<br>DDX6 (MilliporeSigma, Cat# SAB4200837, 1:200)<br>G3BP1 (Santacruz Biotechnology, Cat# sc-365338, 1:200)<br>Actin, phalloidin-488 (ThermoFisher Scientific, Cat# Al2379, 15:200)<br>anti-RBM20 rabbit polyclonal primary antibody (Abeam, ab233147, 1:500)<br>GAPDH (mouse monoclonal [6C5]; Abeam #8245, 1:500)<br>Stabilized goat anti-rabbit hRP conjugated (Pierce, 1858415, 1:1000)<br>Stabilized goat anti-mouse hRP conjugated (Pierce, 1858413, 1:1000)<br>Alexa Fluor 4568 goat anti-rabbit lgG secondary antibody (lnvitrogen, A-11011, 1:100)
Validation	Antibodies were validated in positive and negative control conditions where immuno-activity has been previously reported.<br>Antibodies were also validated from supplier<br>(see catalogue numbers).

Eukaryotic cell lines

Policy information about cell lines

Cell line source(s)	https://www.coriell.org/O/Sections/Search/Sample_Detail.aspx?Ref=GM25256<br>WTB6 (https://www.ncbi.nlm.nih.gov/pmc/articles/PMC4063274/)<br>HS-27A (https://www.atcc.org/products/crl-2496)
Authentication	Sequenced verified (RNA-Seq and exome). iPSC-CMs were differentiated from a sub-culture of the original iPSCs.
Mycoplasma contamination	Verified to have no contamination.
Commonly misidentified lines<br>(See ICLAC register)	no commonly misidentified cell lines were used in the study.

Animals and other organisms

Policy information about studies involving animals; ARRIVE guidelines recommended for reporting animal research

Laboratory animals	NA
Wild animals	NA
Field-collected samples	NA
Ethics oversight	The UCSF Committee on human research #10-02521 approved the study protocol for iPSCs. The human iPSC lines used in this study were generated from a healthy male patient, WTCll (Kreitzer et al., 2013; Miyaoka et al., 2014) using the episomal reprogramming method (Okita et al., 2011). Informed consent was obtained for this procedure.

Note that full information on the approval of the study protocol must also be provided in the manuscript.

Flow Cytometry

Plots

Confirm that:

☒ The axis labels state the marker and fluorochrome used (e.g. CD4-FITC).

☒ The axis scales are clearly visible. Include numbers along axes only for bottom left plot of group (a 'group' is an analysis of identical markers).

☒ All plots are contour plots with outliers or pseudocolor plots.

☒ A numerical value for number of cells or percentage (with statistics) is provided.

Methodology

Sample preparation	Day 15 cells were dissociated to single cells using 0.2 ml of 0.25% Trypsin (UCSF Cell Culture Facility) per well of a 24-well plate with incubation at 37°C. Cells were resuspended in 0.8 ml/well EB medium (Knockout DMEM supplemented with 20% FBS (HyClone), Glutamax (Gibco), Non-essential Amino Acids (UCSF Cell Culture Facility), and 0.1 mM beta-Mercaptoethanol (Sigma)). 200 µl (about 200,000) cells were pelleted and fixed in 4% paraformaldehyde at room temperature for 15 minutes. Cells were permeabilized in FACS Buffer (DPBS without calcium and magnesium, 4% FBS, and 2 mM EDTA (Gibco)) with 0.5% (w/v) saponin (Sigma). Cells were stained with 100 µl of 0.002 µg/µl (1:100) Mouse anti-human cardiac Troponin T (cTnT) primary antibody (Thermo, MS-295-P) in FACS Buffer with saponin at room temperature for 30 minutes and washed. Cells were then stained with 100 µl of 0.01 µg/µl (1:200) Alexa Fluor 488 goat anti-mouse IgG secondary antibody (Invitrogen, A-11029) in FACS Buffer with saponin at room temperature for 30 minutes and washed. Finally, cells were stained with 100 µl of 0.01 µg/µl (1:1000) Hoechst 33342 (Molecular Probes) in FACS buffer at room temperature for 5 minutes and passed through a 0.4 µm filter (Millipore) to remove cell clumps.
Instrument	BD lSRII flow cytometer
Software	Data were collected using the MACSQuant VYB flow cytometer (Miltenyi Biotec) and analyzed using FlowJo.
Cell population abundance	No cell purification by sorting was performed.
Gating strategy	A negative gate was selected for coincident cTnT (FITC-A) and SSC-A positivity in undifferentiated iPSC, with less than 1% of cells in this gate. This same gate was used to quantify cardiomyocytes upon differentiation.

☒ Tick this box to confirm that a figure exemplifying the gating strategy is provided in the Supplementary Information.
